# Dispositional mindfulness and mental health among Chinese college students during the COVID-19 lockdown: The mediating role of self-compassion and the moderating role of gender

**DOI:** 10.3389/fpsyg.2022.1072548

**Published:** 2023-01-11

**Authors:** Dan Zhang, Jianbo Shen

**Affiliations:** ^1^Key Laboratory of Adolescent Cyberpsychology and Behavior of Ministry of Education, Key Laboratory of Human Development and Mental Health of Hubei Province, Central China Normal University, Wuhan, China; ^2^Zhixing College of Hubei University, Wuhan, China; ^3^Collaborative Innovation Center of Assessment Toward Basic Education Quality, Central China Normal University Branch, Wuhan, China; ^4^The National Tax Institute of the STA, Yangzhou, China

**Keywords:** COVID-19 lockdown, dispositional mindfulness, self-compassion, mental health, mediating effect, gender

## Abstract

College students’ mental health has been seriously impacted during the global COVID-19 lockdown. There is evidence that dispositional mindfulness is beneficial to mental health. However, few studies have looked at the relationship between dispositional mindfulness and mental health from the standpoint of self-compassion. Furthermore, it is unclear under what circumstances dispositional mindfulness is linked to mental health during COVID-19 lockdown. To fill this gap, the current study investigated self-compassion as a possible mediating factor and gender as a possible moderating effect between dispositional mindfulness and mental health. The sample included 1,018 Chinese university students during the COVID-19 lockdown (M age = 20.12; SD age = 1.17) who had completed self-report questionnaires on dispositional mindfulness, self-compassion, and mental health. According to the findings of mediation analysis, self-compassion partially mediated the relationship between dispositional mindfulness and mental health. The moderating analysis also revealed significant moderating effects of dispositional mindfulness, self-compassion, and mental health. Male college students’ mental health was significantly better protected, and the buffering effects of dispositional mindfulness and self-compassion on their mental health were significantly stronger than those of female college students. These findings advance our understanding of the process and mechanism between dispositional mindfulness and mental health, broadened and deepened the understanding of the relationship between dispositional mindfulness and mental health, as well as the mediating role of self-compassion and the moderating role of gender, and offer practical guidance for improving college students’ mental health during the COVID-19 lockdown.

## 1. Introduction

Since the outbreak of COVID-19, home isolation, social distancing, and cordoning off public spaces have become common measures to stop the spread of the virus (World Health Organization, 2020). The state of alert for COVID-19 was accompanied by a state of alert for human mental health. Many experts consider the occurrence of mental health problems during COVID-19 to be a parallel pandemic (Mucci et al., 2020). The authors emphasize that many people suffer from depression and anxiety as a result of social isolation and distancing (Hall et al., 2008). More than 70 million people worldwide have suffered from depression and 90 million from anxiety since the outbreak of COVID-19. Previous studies have reported that during the COVID-19 lockdown, approximately a quarter of patients with mental disorders were diagnosed with anxiety at the time of consultation, followed by depression (17.5%) (Ramadan et al., 2022). A meta-analysis of the prevalence of anxiety and depression in the United Kingdom during the COVID-19 lockdown showed that the first COVID-19 lockdown increased the prevalence of anxiety and depression in the general population compared to pre-pandemic data, where a 26.35% increase in anxiety and a 27.88% increase in depression were observed (Dettmann et al., 2022).

According to some studies, the vulnerable age group for general mental health during the COVID-19 outbreak is 21–40 years. Other researchers discovered that participants under the age of 35 had higher levels of anxiety and depression than other age groups (Ahmed et al., 2020; Huang and Zhao, 2020). Furthermore, anxiety and depression levels among students have increased (Wang et al., 2020).

College is a transitional period in life, where students face numerous mental health risks such as academic, financial, and social pressures, increasing responsibility, a desire for success, and support resource conversion (Cooke et al., 2006; Tosevski et al., 2010; Liu et al., 2019). During the COVID-19 lockdown, students faced many issues, such as long-term isolation from others on campus, especially people they care about (Cao et al., 2020), restricted travel, and uncertainty about whether or when the examination would be held. Activities to cope with emotions were limited (such as leaving campus only when necessary, maintaining social distance, prohibiting large gatherings, and so on). They were also concerned about their future academic standing and the difficulty of finding work in the future. This led to great psychological distress and aggravated mental health problems (Wang et al., 2020). During the COVID-19 outbreak, an early study on a sample group of college students showed that college students had higher anxiety levels than the general population (Wang and Zhao, 2020). A survey of college students found that more than half of the students (65.3%) reported low well-being during the pandemic (Dodd et al., 2021). During closed isolation, some people with no history of mental disorders develop depression and anxiety issues (Luykx et al., 2020; Otu et al., 2020).

To better protect students’ mental health, it is critical to pay attention to the protective factors, mediating mechanisms, and regulating mechanisms that affect their mental health during COVID-19 lockdown.

### 1.1. The relation between dispositional mindfulness and mental health

Along with the rise and popularity of positive psychology, researchers widely consider mindfulness as a positive protective factor and a key variable in individual development. Mindfulness impacts individual mental health and well-being, particularly under environmental change, and mindfulness is a strong predictor of individual positive adaptation outcomes. College students face multiple internal and external risks during the COVID-19 lockdown. Mindfulness is a positive psychological resource and trait, which is considered an important protective factor in the development of life and various outcomes (Huang et al., 2021b). Mindfulness can increase positive qualities such as awareness, insight, wisdom, compassion, and serenity (Kabat-Zinn, 2000; Goldstein, 2002), which protects mental health. Therefore, college students should be cared for and supported to overcome the COVID-19 crisis by focusing on their mindfulness (Tran et al., 2022). Some scholars have argued that mindfulness is a trait that improves with practice or experience. According to their findings, there are four types of mindfulness: traits, states, training, and treatment. Mindfulness was viewed as a stabilizing quality in this study (Chiesa, 2013; Good et al., 2016). Dispositional mindfulness is defined as a person’s ability and proclivity to maintain mindfulness in daily life, to adopt a nonjudgmental and accepting attitude, and to focus on thoughts and feelings in the present moment (Brown and Ryan, 2003). They are extremely important psychological resources for helping people cope with stress and adversity (Dixon and Overall, 2016), and people develop long-term changes in cognitive, emotional, or behavioral tendencies (Bajaj et al., 2016).

In isolation conditions, one of the most important indicators of individual health is mental health, which is defined as a state of optimal human functioning beyond the experience of well-being [for example, Ryff and Keyes, 1995]. Because mindfulness is achieved by focusing attention on immediate experience, people recognize that thoughts and emotions are mental events, not facts, and can change mental habits such as anxiety and depression. Recent research has reported that dispositional mindfulness is negatively associated with anxiety and depression (Lam et al., 2020) and can effectively improve individual subjective well-being and mental health (Nyklíček et al., 2011).

According to the Dual-factor Model of Mental Health, when describing the level of young people’s mental health, the assessment indicators should include both positive subjective well-being and negative psychopathological symptoms (Greenspoon and Saklofske, 2001; Eklund et al., 2010). Among the positive indicators, the predecessors used life satisfaction to assess subjective well-being (Diener et al., 1985; Huppert, 2009). Satisfaction with life is a standard to evaluate individual positive psychological status as a core index to measure individual happiness and quality of life (Cowen, 1994). Anxiety and depression have been the most commonly used negative psychological indicators in previous research (American Psychiatric Association, 2000; Greenspoon and Saklofske, 2001; Merrell, 2008; Eklund et al., 2010; Hides et al., 2020). This study assessed people’s mental health using life satisfaction, anxiety and depression (Wang et al., 2016).

### 1.2. The mediating role of self-compassion

Self-compassion has been described as being kind to oneself in the face of difficulties and failures, being able to accept one’s shortcomings and deficiencies, being able to assess oneself objectively, and being kind and caring when one is sad and depressed (Neff, 2003a, 2009).

Some studies have reported that mindfulness and self-compassion are positively correlated, with mindfulness being a source and inner exploration of self. People who have high levels of self-compassion have a more balanced and objective view of negative life events, so they often experience fewer negative emotions (Neff, 2009). When people with high self-compassion face their own shortcomings, they tend to adopt a friendly and enthusiastic manner that can enhance their positive emotional and subjective well-being (Hollis-Walker and Colosimo, 2011). Self-compassion has been shown to have a positive impact on the mental health and well-being of adults (e.g., nurses) COVID-19 (Gerace, 2022).

### 1.3. The moderating effect of gender

A cohort study from Saudi Arabia showed that being female during the COVID-19 lockdown was significantly correlated with a higher likelihood of Emergency Department (Ed) visits for mental health disorders compared to male dominance before the COVID-19 pandemic (Ramadan et al., 2022). During the COVID-19 pandemic, female college students are more at risk for symptoms of depression and anxiety, and their negative emotions are positively associated with depression and anxiety (Carlucci et al., 2020; Mazza et al., 2020). Previous research has found a link between gender, dispositional mindfulness, and comorbid symptoms of psychopathology, with males having a higher level of dispositional mindfulness than females in a sample of undergraduates (Wang and Chopel, 2020). Subgroup analyses revealed that female college students had a higher overall morbidity of depressive symptoms than male college students, and there were differences in the morbidity of anxiety and depressive symptoms in college students from different countries (Chang et al., 2021).

According to previous research on self-compassion and adolescent development, as adolescents undergo significant physiological changes, there are significant gender differences in self-compassion, with female adolescents exhibiting lower levels of self-compassion than male adolescents of the same age. Gender is associated with an increase in psychopathological symptoms such as anxiety and depression (Copeland et al., 2011). Gender moderates the relationship between self-compassion and anxiety and depression symptoms, and self-compassion has a greater protective effect on anxiety and depression in male college students than in female college students (Galla, 2016).

## 2. The present study

**Figure 1:**
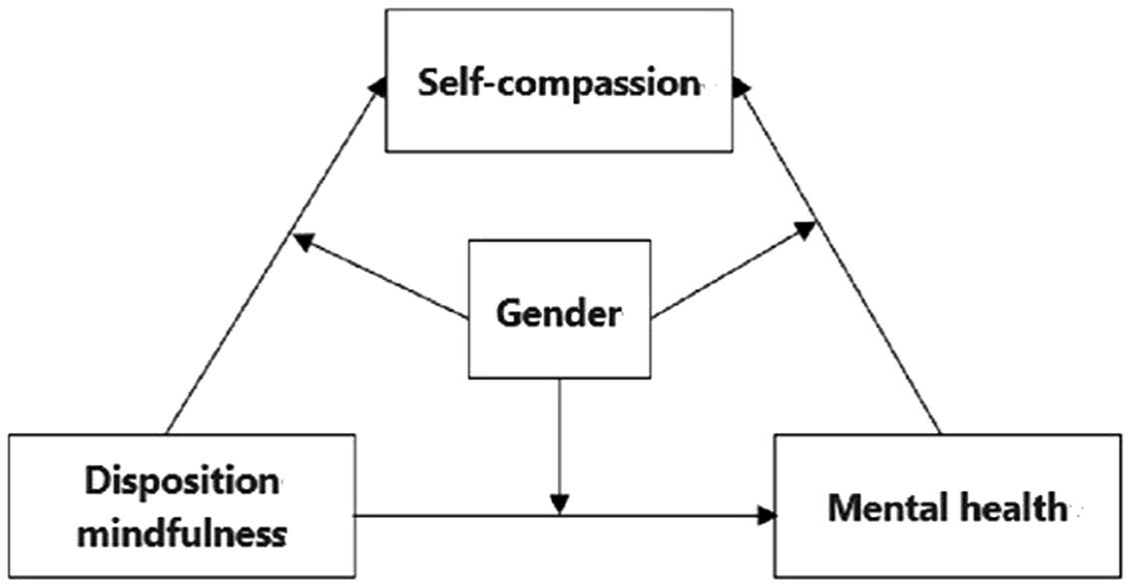
The proposed conceptual model.

Previous research on the mental health of college students during the COVID-19 lockdown found that mindfulness is a predictor of mental health and a protective factor against stress, but empirical research on the mediating and moderating mechanisms was lacking. In this study, we hypothesized that there is a moderated mediation model in which dispositional mindfulness and self-compassion are predictors of subjective well-being, self-compassion has a mediating effect between dispositional mindfulness and mental health. Gender plays a moderating role between dispositional mindfulness, self-compassion, and mental health (Figure 1).

*Hypothesis 1*: The relationship between dispositional mindfulness and mental health is mediated by self-compassion; that is, dispositional mindfulness is positively related to self-compassion, and self-compassion is positively related to mental health.

*Hypothesis 2*: Gender has a moderating effect on the direct relationship between dispositional mindfulness and mental health, i.e., male college students' dispositional mindfulness correlates more strongly with mental health than female college students.

*Hypothesis 3*: Gender will moderate the indirect effect of mindfulness on mental health; that is to say, male college students have a stronger correlation between dispositional mindfulness and self-compassion, and male college students have a stronger correlation between self-compassion and mental health. The effect of dispositional mindfulness and self-compassion on mental health is greater in male college students than in female college students.

## 3. Materials and methods

### 3.1. Participants and procedure

#### 3.1.1. Participants

We chose a full-time target university in the lockdown area for participant recruitment for the limited lockdown areas in China in September 2022. We invited 1,248 students from the target university’s department of social science and polytechnic to participate in the online survey to ensure a diverse sample. The school’s program administrator, who keeps track of the students’ enrollment, assists with the recruitment process. Students were instructed not to distribute online survey links to others. Given the low response rate of online surveys, an incentive of 8 RMB (about 1.1 USD) was provided for participation. The test period was late September 2022, during an outbreak of COVID-19 omicron virus infections in Wuhan, China. The government has imposed a strict and ongoing lockdown on this university since the beginning of the semester to prevent the spread of the COVID-19 pandemic. Every college student was required to study, stay in school, and take nucleic acid tests every other day. This lockdown lasted 4 weeks. By early October 2022, 1,025 students had completed and submitted the survey. We had a final analytic sample of 1,018 college students after excluding 7 cases due to incomplete answers. The response rate was 82%.

#### 3.1.2. Procedure

This study was approved by the Ethics Committee for Psychological Research of the corresponding author’s institution. We used questionnaires with high reliability and validity, which are widely used. The wording of some questions was changed to motivate participants to give accurate answers (MacKenzie and Podsakoff, 2012). Participants indicated their informed consent before the data collection by ticking a box on the informed consent item. The consent form included a brief description of the research project. The participants were informed that their provided information will be kept confidential and anonymous. Their responses would be used only for research purposes, and that they could withdraw at any time without penalty. The questionnaires were completed in about 25 min by the participants.

### 3.2. Measures

#### 3.2.1. Dispositional mindfulness

Dispositional mindfulness was measured using the short form of FFMQ-SF (Bohlmeijer et al., 2011), a reliable and valid instrument that supports the multi-faceted structure of mindfulness as previously proposed (Baer et al., 2006). In the current sample of college students, we performed the confirmatory factor analysis (CFA) to examine the validity of the construct. The index of CFA of this scale showed a good fit: *χ*^2^/df = 1.759, RMSEA = 0.027, CFI = 0.989, TLI = 0.988 SRMR =0.021. The scale includes 24 items, acting with awareness (e.g., I cannot pay attention to the things I am doing; reverse scoring; *α* = 0.904); non-judgmental (e.g., I judge my own thoughts as good or bad; reverse scoring; *α* = 0.912); non-responding (e.g., I observe my feelings without getting lost in them; *α* = 0.906); description (e.g., I naturally like to put my experiences into words; *α* = 0.909); observation (e.g., I pay attention to the things like the wind in my hair or sunshine on my face; *α* = 0.882). Cronbach’s α of the entire scale was 0.950. A 7-point Likert scale (1 = never, 7 = always) was used.

#### 3.2.2. Self-compassion

The Chinese version of Neff’s Self-Compassion Scale was used to assess self-compassion (Neff, 2003b). The scale and its Chinese translation have been used in cross-cultural studies, and their reliability and validity have been confirmed (Neff, 2003b; Neff et al., 2018; Huang L. et al., 2021). The CFA indicated that this scale has a good fit: *χ*^2^/df = 1.687, RMSEA = 0.026, CFI = 0.988, TLI = 0.987， SRMR = 0.019. It contains 26 elements and six factors: self-kindness, shared humanity, and mindfulness; self-cold, isolation, and over immersed. Two example questions are: “I try to see my failings as part of the human condition” and “When I’m feeling down, I obsess to fix everything that’s wrong.” In the current sample, Cronbach’s α of the entire scale was 0.947 and of the individual subscales was 0.903, 0.876, 0.883, 0.899, 0.882, and 0.882. Items are rated on a 7-point Likert scale from 1 (never) to 7 (always).

#### 3.2.3. Mental health

##### 3.2.3.1. Satisfaction with life

We used the Satisfaction with Life Scale (Pavot and Diener, 2008) to measure the participants’ life satisfaction. The SWLS is a widely used, well-validated, and concise instrument for measuring general well-being with five items. Participants rate items (for example, “I am satisfied with my life”) on a 7-point Likert scale, indicating how much they agree or disagree with each statement. Higher scores indicate higher levels of life satisfaction. The CFA indicated that this scale provides an acceptable fit: *χ*^2^/df = 7.355, RMSEA = 0.079, CFI = 0.989, TLI = 0.979 SRMR = 0.016. Cronbach’s α is equal to 0.899.

##### 3.2.3.2. Anxiety and depression

The Hospital Anxiety and Depression Scale (HADS) was developed by Zigmond AS and Snaith RP in 1983 (Zigmond and Snaith, 1983). The HAD consisted of 14 items, of which 7 items were rated with depression and 7 with anxiety. There were six reversed questions, five on the depression subscale and one on the anxiety subscale. Lower scores indicate better levels of anxiety and depression in life. The CFA indicated that this scale provides an acceptable fit: *χ*^2^/df = 2.370, RMSEA = 0.037, CFI = 0.989, TLI = 0.987 SRMR =0.016. The Cronbach’s α of the total scale was 0.902, and each subscale was 0.922, 0.927.

The CFA of satisfaction with Life and anxiety and depression showed a good fit to the data: *χ*^2^/df = 1.999, RMSEA = 0.031, CFI = 0.988, TLI = 0.986, SRMR = 0.020. The total average score for mental health was calculated by principal component analysis.

## 4. Data analysis

We first used descriptive statistics and correlation analysis for variables of interest to analyze the survey data. Second, the SPSS macro-PROCESS (Hayes, 2013) was conducted to investigate further the mediating effect of self-compassion and the moderating effect of female/male in the correlation between dispositional mindfulness and mental health. As age, grade, single child status, residential location (Sedlmeier et al., 2012; Homan, 2016; Bluth et al., 2017; López et al., 2018; Wang et al., 2021), and meditation (Kabat-Zinn, 1994; Soler et al., 2014; Bergomi et al., 2015) of college students have been linked to dispositional mindfulness, self-compassion, and mental health, we controlled for the potential influence of these constructs in the data analysis. In addition, the results of Harman’s univariate test showed that there were 14 different factors with eigenvalues greater than 1 and that the largest factor currently accounted for 28.984% of the variance (threshold level & LT; 35%) to check for possible bias from the common method to test. Therefore, there is no overall method bias in this study.

## 5. Results

### 5.1. Descriptive statistics and correlational analyses

This sample contained 34% males and 66% females. Participants ranged in age from 17 to 24 years (M-age = 20.12; SD-age = 1.17), with 4.2% in freshmen, 15.6% in sophomores, 44.1% in juniors, and 36.1% in seniors (see Table 1).

**Table 1:** Demographic information.

Sample characteristics	Type	No.	Percentage
Gender	Female	672	66.0
	Male	346	34.0
Single child status	Yes	385	37.8
	No	633	62.2
Age	17.00	8	0.8
	18.00	69	6.8
	19.00	227	22.3
	20.00	335	32.9
	21.00	265	26.0
	22.00	97	9.5
	23.00	14	1.4
	24.00	3	0.3
Grade	Freshman	43	4.2
	Sophomore	159	15.6
	Junior	449	44.1
	Senior	367	36.1
Residence location	City	329	32.3
	Rural	689	67.7
Meditation	No	226	22.2
	Yes	792	77.8

Pearson’s correlation coefficient matrix, mean, and standard deviation of all variables are shown in Table 2. Pearson’s correlation coefficient test revealed that dispositional mindfulness was significantly positively correlated with self-compassion and mental health (*p* < 0.01). Further, there was a significant positive correlation between self-compassion and mental health (*p* < 0.01). It can be seen that the relationship between variables in this study has been preliminarily confirmed, and the research hypothesis can be further tested.

**Table 2:** Correlation analysis of variables.

Variables	*M*	SD	DM	SC	MH
DM	4.333	0.893	1.000		
SC	4.440	0.855	0.472**	1.000	
MH	4.779	1.217	0.478**	0.591**	1.000

*N* = 1,018. M, mean; SD, standard deviation; DM, dispositional mindfulness; SC, self-compassion; MH, mental health, **p < 0.01.

### 5.2. Testing for the mediation model (self-compassion as mediator)

Table 3 testing the mediation role of self-compassion.

**Table 3:** Testing the mediation role of self-compassion.

Criterion	Predictors	*β*	SE	*t*	*R*	*R* ^2^	Adjusted *R*^2^	*F*
MH								
	Age	−0.014	0.014	−1.044***	0.511	0.261	0.256	59.453***
	Grade	−0.011	0.019	−0.560				
	Single child status	0.024	0.033	0.734				
	Residence location	−0.161	0.035	−4.653***				
	Meditation	0.161	0.039	4.121				
	DM	0.288	0.018	15.811***				
SC								
	Age	0.073	0.020	3.611	0.506	0.256	0.252	58.111***
	Grade	−0.010	0.028	−0.351				
	Single child status	0.017	0.048	0.344				
	Residence location	−0.230	0.051	−4.508***				
	Meditation	0.093	0.057	1.620***				
	DM	0.414	0.027	15.451***				
MH								
	Age	−0.037	0.012	−3.022**	0.646	0.417	0.413	103.130***
	Grade	−0.008	0.017	−0.449				
	Single child status	0.019	0.029	0.648				
	Residence location	−0.090	0.031	−2.877**				
	Meditation	0.132	0.035	3.795***				
	DM	0.159	0.018	8.818***				
	SC	0.312	0.019	16.438***				

*N* = 1,018. DM, dispositional mindfulness; SC, self-compassion; MH, mental health, **p < 0.01, ***p < 0.001.

First, Hayes’ Model 4 of the PROCESS program was used to examine the mediating effect of self-compassion between dispositional mindfulness and mental health (Hayes, 2013). Taking into account covariates such as residential location, age and grade, status as an only child, meditation or not, the table revealed that dispositional mindfulness scores positively predicted self-compassion (*β* = 0.414, *t* = 15.451, *p* < 0.001) and mental health (*β* = 0.288, *t* = 15.811, *p* < 0.001). Controlling the independent variable dispositional mindfulness, self-compassion positively predicted mental health (*β* = 0.312, *t* = 16.438, *p* < 0.001). Hypothesis 1 is tested.

Second, Table 4 demonstrated that self-compassion partially mediates the effect between dispositional mindfulness and mental health, since both the direct effect of dispositional mindfulness on mental health and the mediating role of self-compassion did not contain 0 within Bootstrap 95% confidence intervals. These direct (0.159) and mediating (0.129) roles accounted for 55 and 45% of the total effect (0.288), respectively. Hypothesis 2 is tested.

**Table 4:** Mediation effect of self-compassion.

Mental health	Effect	BootSE	BootLLCI	BootULCI	Effect percentage
Total effect of DM	0.288	0.018	0.252	0.324	
Direct effect of DM	0.159	0.018	0.123	0.194	55.11%
Mediate effect of SC	0.129	0.013	0.106	0.156	44.89%

*N* = 1,018. DM, dispositional mindfulness; SC, self-compassion; MH, mental health.

### 5.3. Testing for gender as moderator

In the study, to test a moderated mediation model, we run the PROCESS macro (Model 59) (Hayes, 2013) to test the moderating effect of self-compassion. After gender was included in the model, dispositional mindfulness significantly predicted self-compassion (*β* = 0.418, *t* = 15.898, *p* < 0.001) (Table 5). The interaction term between dispositional mindfulness and gender (a × b) was a significant positive predictor of self-compassion (*β* = 0.353, *t* = 6.530, *p* < 0.001). Dispositional mindfulness was a significant positive predictor of mental health (*β* = 0.144, *t* = 7.746, *p* < 0.001), as was self-compassion (*β* = 0.312, *t* = 15.826, *p* < 0.001). The interaction term (a × b) between dispositional mindfulness and gender was an important predictor of mental health with significant predictive effects (*β* = 0.108, *t* = 2.499, *p* < 0.05). The interaction term between gender and self-compassion (b × c) is a significant predictor of mental health (*β* = 0.198, *t* = 4.382, *p* < 0.001).

**Table 5:** Testing the gender as a moderator.

Criterion	Predictors	*β*	SE	*t*	*R*	*R* ^2^	Adjusted *R*^2^	*F*
SC					0.536	0.287	0.282	50.873***
	Age	0.075	0.020	3.784***				
	Grade	0.000	0.028	−0.007				
	Single child status	0.029	0.047	0.619				
	Residence location	−0.227	0.050	−4.539***				
	Meditation	0.063	0.056	1.112				
	DM	0.418	0.026	15.898***				
	Gender	−0.064	0.048	−1.322				
	Gender*DM(a × b)	0.353	0.054	6.530***				
MH	Age				0.673	0.454	0.448	83.562***
	Grade	−0.036	0.012	−2.973**				
	Single child status	−0.002	0.017	−0.100				
	Residence location	0.024	0.028	0.858				
	Meditation	−0.088	0.030	−2.916**				
	Age	0.112	0.034	3.309**				
	DM	0.144	0.019	7.746***				
	SC	0.312	0.020	15.826***				
	Gender	0.009	0.029	0.324				
	Gender*DM(a × b)	0.108	0.043	2.499*				
	Gender*SC(b × c)	0.198	0.045	4.382***				
Conditional indirect effect analysis				Effect	BootSE	BootLLCI	BootULCI	
		Female		0.073***	0.012	0.051	0.098	0.073***
		Male		0.288***	0.022	0.247	0.331	0.288***
		Index		0.215***	0.024	0.170	0.263	0.215***

*N* = 1,018. DM, dispositional mindfulness; SC, self-compassion; MH, mental health, *p < 0.05, **p < 0.01, ***p < 0.001.

The indirect effects of mindfulness on self-compassion and self-compassion on mental health in all genders (see Table 5) are both significant. For females, the 95% confidence interval does not include 0 [0.051, 0.098], and the effect value was 0.073; for males, the 95% confidence interval does not contain 0 [0.247, 0.331], and the effect value was 0.288. Furthermore, the difference in indirect effects between high and low does not contain 0, indicating that the mediating effect is significant. These findings indicated that gender is enhanced by mindfulness and the indirect role of self-compassion on mental health, and when gender is male, the mediating effect is more substantial. Hypothesis 3 is tested.

**Figure 2:**
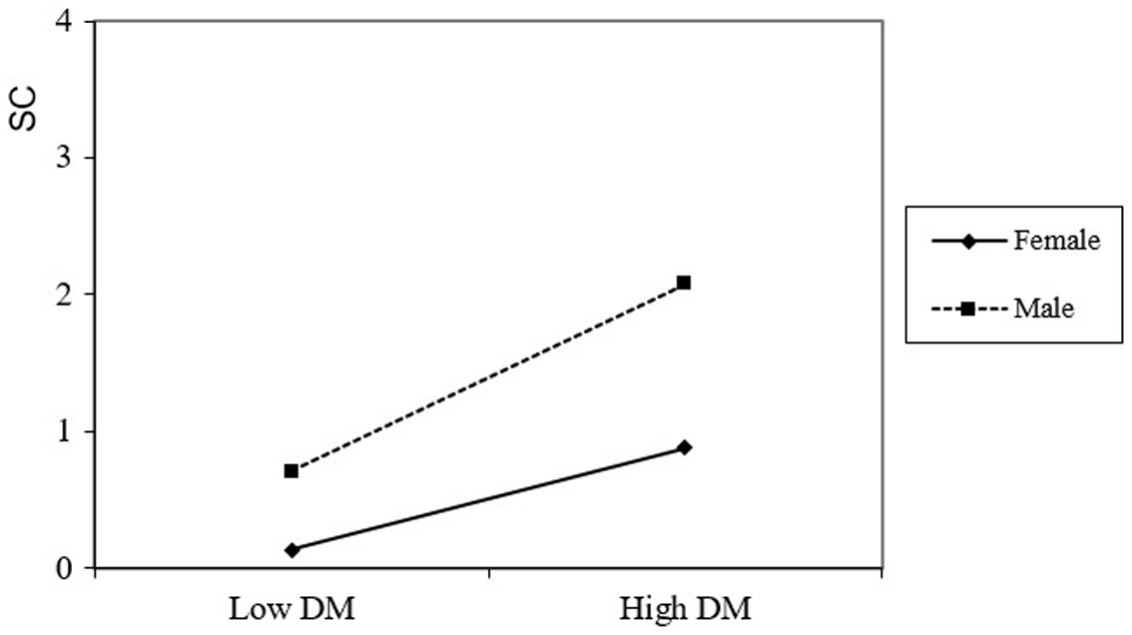
The moderating effect of gender in the association between dispositional mindfulness and self-compassion.

The values of the direct effect of dispositional mindfulness, the values of the mediating effect of self-compassion, and the bootstrap 95% confidence intervals are shown in Table 6. The dispositional mindfulness value has an important positive predictive effect on self-compassion in male students (*β* = 0.650, *t* = 14.581, *p* < 0.001). The female students’ dispositional mindfulness score had an important positive predictive effect on self-compassion (*β* = 0.298, *t* = 9.360, *p* < 0.001), but the female students’ self-compassion increased at a slower rate as their dispositional mindfulness scores increased than males (see Figure 2).

**Table 6:** Effects under different gender conditions.

Total effect	Gender	Effect	SE	*t*	*p*	LLCI	ULCI
DM-MC	Female	0.298	0.032	9.360	0.000	0.235	0.360
	Male	0.650	0.045	14.581	0.000	0.563	0.738
DM-MH	Female	0.108	0.020	5.351	0.000	0.068	0.147
	Male	0.215	0.038	5.635	0.000	0.140	0.290
SC-MH	Female	0.245	0.021	11.664	0.000	0.204	0.286
	Male	0.443	0.040	10.967	0.000	0.364	0.522

*N* = 1,018. DM, dispositional mindfulness; SC, self-compassion; MH, mental health.

**Figure 3:**
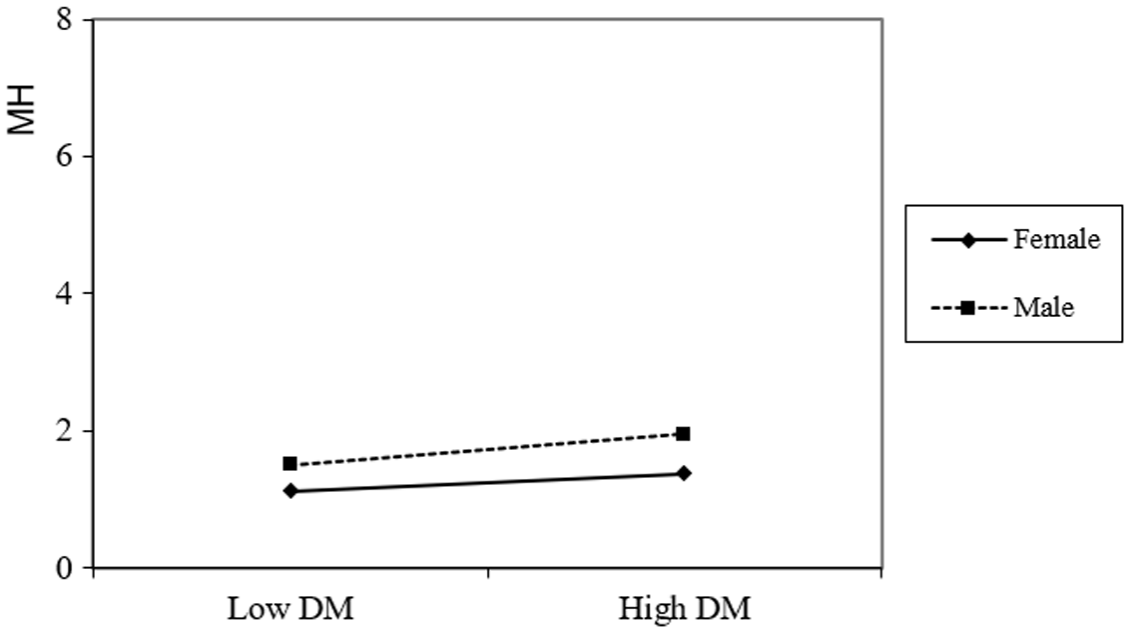
The moderating effect of gender in the relationship between dispositional mindfulness and mental health.

Dispositional mindfulness had a significant positive predictive role on male students’ mental health (*β* = 0.215, *t* = 5.635, *p* < 0.001). Simultaneously, dispositional mindfulness in females was a significant positive predictor of mental health (*β* = 0.108, *t* = 5.635, *p* < 0.001). However, as dispositional mindfulness increased, females’ mental health scores increased more slowly than those of male college students (see Figure 3).

**Figure 4:**
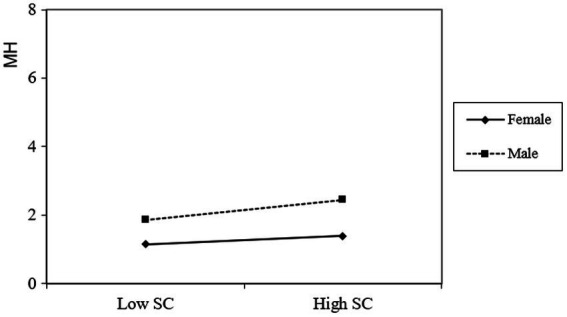
The moderating effect of gender on the association between self-compassion and mental health.

The positive predictive role of self-compassion on the mental health of male students is significant (*β* = 0.443, *t* = 10.967, *p* < 0.001), and self-compassion of female students has a significant positive predictive role on mental health (*β* = 0.245, *t* = 11.664, *p* < 0.001). However, as self-compassion increased, females’ mental health improved more slowly than those of male college students (see Figure 4).

In general, gender has a significant moderating effect on dispositional mindfulness, self-compassion, and mental health. This observation suggests that dispositional mindfulness and self-compassion have a stronger protective and buffering effect in male college students than in females during the COVID-19 lockdown.

## 6. Discussion

The positive role of mindfulness in mental health has garnered widespread attention and empirical support during the COVID-19 pandemic (Demirdogen et al., 2022; Goldberg et al., 2022). This work extends previous studies by demonstrating the linking mechanisms between dispositional mindfulness and mental health. Recent research suggests that self-compassion mediates the relationship between dispositional mindfulness and mental health. Gender influences the relationship between the three, with males having a stronger influence than females. These findings suggest that mindfulness and self-compassion are potential mechanisms for mental health during COVID-19 lockdown, and that male students are better at dispositional mindfulness, self-compassion, and mental health than females, while females require more psychological support. This study uncovered the potential mechanism of dispositional mindfulness and self-compassion affecting mental health and provided a reference for developing targeted mental health intervention programs during the COVID-19 lockdown.

### 6.1. The effect of dispositional mindfulness on mental health

There is ample evidence that dispositional mindfulness is a positive and protective resource for medicine, health care, and psychology (Batchelor, 1994), as it is associated with greater subject well-being and involves less depression and anxiety (Brown and Ryan, 2003). Furthermore, our findings support the findings of systematic reviews and meta-analyses that show mindfulness significantly affects depressive and anxiety symptoms (Strauss et al., 2014; Chu et al., 2018). Dispositional mindfulness has also been found to be a good predictor of self-compassion, depression, anxiety, and subjective well-being in nonclinical populations under COVID-19 lockdown conditions (Conversano et al., 2020). Regarding the relationship between mindfulness and mental health, our results support the Buddhist theory of mindfulness (Batchelor, 1994; Shapiro et al., 2006; Brown et al., 2007), which as a traditional Buddhist teaching, is the core intention of mindfulness. It is believed that mindfulness assists in cultivating the loving mind or bodhicitta and transforming unhealthy and unskilled traits into wholesome and skillful traits for the benefit of all beings (Gethin, 1992/2001). It has also been found that high levels of optimism mindfulness correlate with high levels of self-compassion (Duarte and Pinto-Gouveia, 2017); also, mindfulness can effectively improve individual self-compassion (Eklund et al., 2010), stabilize individual attention and calm mind (called Shamatha in Sanskrit; Bodhi, 2011), and achieve true inner transformation by deliberately choosing and focusing on positive mental states to promote happiness and achieve mental health (Lama and Cutler, 1998). Therefore, universities and psychotherapists can use dispositional mindfulness as an important tool to assist students in combating potential anxiety and depression and increasing happiness during the COVID-19 pandemic (González-García et al., 2021; Huang et al., 2021a).

### 6.2. The mediating role of self-compassion

Previous interventions have shown that mindfulness treatment can promote mental health, suggesting that mindfulness-targeted treatment is most useful in epidemics or crises. Individuals’ level of self-compassion is related to their mental health. Individuals with high levels of self-compassion will improve their defense mechanisms and be better at motivating their positive behavior (Pace et al., 2009). Based on previous studies, this research illustrated the mediating effect of self-compassion on dispositional mindfulness toward mental health. It confirmed the protective role of individual self-compassion and dispositional mindfulness on mental health during the COVID-19 lockdown. Therefore, self-compassion training may be a useful intervention strategy for maintaining mental health. According to our findings, self-compassion improves subjective well-being, is associated with higher life satisfaction, and reduces symptoms of depression and anxiety (Bluth and Blanton, 2015), providing an optimistic link to subjective well-being (Baer et al., 2012). Our findings add to the growing body of evidence that self-compassion mediates the relationship between dispositional mindfulness and mental health (Voci et al., 2019), and that increased self-compassion can help to demonstrate the role of mindfulness in mental health. In other words, self-compassion, consistent with our hypothesis, is an important mechanism explaining why and how mindfulness is associated with better mental health, and self-compassion plays an important role in mental health protection (Neff and Pommier, 2013). Students with a high degree of self-compassion will improve their defense mechanisms, motivate positive behaviors (Pace et al., 2009) and adopt a more positive coping style (Leary et al., 2007) when faced with the lockdown. College students with high levels of self-compassion tend to confront their shortcomings with friendliness and warmth, and it may improve an individual’s subjective well-being (Hollis-Walker and Colosimo, 2011). They also understand negative life events more objectively and use positive coping strategies to reduce anxiety and depression (Van Dam et al., 2011; Neff et al., 2018). College students with low self-compassion will be overly immersed in coronavirus-induced stress due to their numbness and loneliness associated with criticism, depression, anxiety, and other negative psychological symptoms (Van Dam et al., 2011), leading to increased unhappiness and negative psychological symptoms.

Overall, our findings confirm our predictions. Individuals with dispositional mindfulness have a higher degree of self-compassion and tend to have higher subject well-being (Schutte and Malouff, 2011; Chen et al., 2017; Liang et al., 2022), but it is negatively related to anxiety and depression (Dillard and Meier, 2021). As expected, self-compassion mediated the relationship between dispositional mindfulness and well-being. Whether or not college students meditate, the mechanisms between dispositional mindfulness, self-compassion, and subjective well-being were equally significant. This suggests that dispositional mindfulness results from a complex interaction between explicit mindfulness training, genetic predisposition, and the environment in which personality develops (Brown et al., 2007). According to the study, people with high dispositional mindfulness scores included both meditators and non-meditators (whose dispositional mindfulness is genetically inherited). Individuals born with dispositional mindfulness may have better mental health, and self-compassion training is more likely to improve their mental health during COVID-19. Another group of college students born with less dispositional mindfulness may benefit from mindfulness and self-compassion training together, such as mindfulness self-compassion programs of the second generation mindfulness intervention.

### 6.3. The moderating role of gender

It is well known that males and females have different mental health problems. Females tend to have more internalizing disorders than males and a higher prevalence of depression and anxiety (Rosenfield and Mouzon, 2013). There is no doubt that females are more likely to experience mental distress during the COVID-19 lockdown. To begin, our research found that gender has a moderating effect on the relationship between mindfulness and well-being and depression and anxiety in college students. Female college students are more socially sensitive than males and are more likely to contemplate a coping strategy, which leads to increased mental health symptoms (Neff, 2003b; Yuan et al., 2010; Van Droogenbroeck et al., 2018). In other words, female college students have a stronger negative correlation between dispositional mindfulness and psychological symptoms of anxiety and depression and a weaker positive correlation with well-being (Webb et al., 2021a,b). Females are more likely to experience mental distress due to the uncertainty of the epidemic, infection risk, and various stresses and shocks. Their dispositional mindfulness increases more slowly than male college students, as does their well-being. Therefore, during the COVID-19 lockdown, females with low dispositional mindfulness warrant more attention, support, and protection in the mental health education and maintenance of college students. Second, the study also found that gender plays a moderating role in the relationship between dispositional mindfulness and self-compassion in college students. With the increase in dispositional mindfulness, college students’ self-compassion will increase, but that of female students will increase more slowly than male college students; that is, when the COVID-19 lockdown brings life and psychological discomfort, female students are more prone to self-criticism and being isolated. Males have greater self-compassion and common humanity, and they recover faster. That is, gender not only modifies college student attitudes toward mindfulness and self-compassion, but it also modifies the relationship between self-compassion and mental health. Compared to the protective effects of self-compassion on the mental health of male college students, the increase in well-being in female college students is slower, and the decrease in anxiety and depression is slighter during the increase in self-compassion.

## 7. Limitations and future directions

Although we have conducted a comprehensive study of the correlation between dispositional mindfulness and mental health, some shortcomings remain. First of all, this study adopts a cross-sectional study design, and experimental studies or longitudinal studies with more causal inferential power are needed to investigate the causal relationship further. Second, limited by the research funding, this study failed to analyze a sample across the country. Only selected students from a university in one city were recruited as a research sample. This limits the generalization of the results to some extent; future studies can be conducted on a larger scale to improve the application of the findings. Because this study was conducted among Chinese college students, extrapolating our findings to other age groups or populations in different countries may pose several challenges. Furthermore, no participants with a diagnosed psychiatric disorder were recruited for the current study. Therefore, the results can only be generalized to other community samples. More research should test a moderated mediation model in more diverse samples. Third, the disproportionate gender composition, with more females than males in the sample, may confound the correlation we examined. Furthermore, mindfulness is not the only reason for mental health. Future research could incorporate multiple factors to protect college students during the COVID-19 lockdown.

## 8. Conclusion

Despite these limitations, the current study sheds light on how and in what state mindfulness is related to mental health during COVID-19 Lockdowns and may help inform intervention strategies to reduce depression and anxiety. First, our findings indicate that increasing dispositional mindfulness in college students during lockdown could effectively reduce anxiety and depression while also improving subjective well-being. Numerous studies have found that the university provides online or offline programs such as mindfulness-based stress reduction, mindfulness-based cognitive therapy, dialectical behavior therapy, acceptance and attachment therapy, and others that can help college students develop and improve their dispositional mindfulness. Then, dispositional mindfulness can alleviate anxiety and depression and protect well-being through self-compassion. The self-compassion intervention may have a better intervention effect in people with high scores for dispositional mindfulness. For those with natural dispositional mindfulness, self-compassion training may be more appropriate to improve their mental health. Other college students can improve their mental health by practicing mindfulness and self-compassion together. Third, gender influences mindfulness, self-compassion, and mental health. For female college students, improving dispositional mindfulness is particularly important for improving self-compassion and mental health. Therefore, strengthening dispositional mindfulness and self-compassion interventions for females is a reliable treatment to alleviate symptoms of anxiety and depression and protect the subject’s well-being during the COVID-19 lockdown.

## Data availability statement

The raw data supporting the conclusions of this article will be made available by the authors, without undue reservation.

## Ethics statement

This study was approved by the Ethics Committee for Psychological Research of the corresponding author’s institution. Written informed consent from the participants was not required in accordance with the national legislation and institutional requirements.

## Author contributions

DZ: methodology, formal analysis, writing – original draft, writing – review and editing, and investigation. JS: translation, polishing, revised validation, and supervision. All authors have read and agreed to the published version of the manuscript.

## Funding

This study was supported by the Research Program Funds of the Collaborative Innovation Center of Assessment toward Basic Education Quality (2021–04–014-BZPK01), Open Project of Key Laboratory of Adolescent Cyberpsychology and Behavior (Central China Normal University), Hubei Province Key Laboratory of Human Development and Mental Health Ministry of Education (2019B07), and Self-Determined Research Funds of Central China Normal University from the Colleges Basic Research and Operation of Ministry of Education (CCNU19TS076).

## Conflict of interest

The authors declare that the research was conducted in the absence of any commercial or financial relationships that could be construed as a potential conflict of interest.

## Publisher’s note

All claims expressed in this article are solely those of the authors and do not necessarily represent those of their affiliated organizations, or those of the publisher, the editors and the reviewers. Any product that may be evaluated in this article, or claim that may be made by its manufacturer, is not guaranteed or endorsed by the publisher.
